# Successful macro-replantation of traumatic upper extremity amputation and type C-spinal cord injury with good functional recovery – a case report

**DOI:** 10.1016/j.ijscr.2025.111570

**Published:** 2025-06-26

**Authors:** Christian Thomas Hübner, Victor Sander, Thomas Meszaros, Hans-Christoph Pape, Nicole Lindenblatt, Christian Hierholzer

**Affiliations:** a Department of Traumatology, University Hospital Zurich, University of Zurich, Raemistrasse 100, 8091 Zurich, Switzerland; b Department of Plastic Surgery and Hand Surgery, University Hospital Zurich, University of Zurich, Raemistrasse 100, 8091 Zurich, Switzerland; c Department of Orthopedic surgery, Hospital of Fribourg, Fribourg, Switzerland

**Keywords:** Amputation, Macroreplantation, Polytrauma, Spine injury, Case report

## Abstract

**Introduction:**

Traumatic amputation and unstable spine injury both require timely interventions to achieve best outcome. A young polytrauma patient with upper extremity amputation and spinal cord injury is presented. The patient underwent macro-replantation of the upper extremity and spinal canal decompression and dorsal stabilization.

**Case presentation:**

A male patient sustained polytrauma from a train accident with traumatic amputation of the right forearm. Diagnostic work-up revealed severe thoracic injuries and an open translocation injury type C L1/L2. The morphology was suggestive of complete paraplegia resulting in wheelchair-dependency.

After initial treatment according to ATLS™ guidelines, replantation of the upper extremity was achieved within 5 h. A 30-degree elbow arthrodesis was established for future wheelchair mobility. Spinal cord injuries were addressed using dorsal spinal stabilization and decompression, followed by ventral spondylodesis.

The patient achieved remarkable good functional recovery, demonstrating walking mobility and performing active wrist flexion and extension of the replanted extremity.

**Discussion:**

Treatment of polytrauma patients is a challenging situation for healthcare providers.

Acute life-threatening injuries must be addressed before peripheral injury patterns are treated. We describe rational and individual decision-making process to attempt macro-replantation of an upper extremity amputation in a polytrauma patient with additional dislocation fracture of the lumbar spine.

**Conclusion:**

In this rare case, an excellent recovery after severe spinal cord injury and macro-replantation of traumatic amputation of the upper extremity is reported. Indication for macro-replantation of the upper extremity was an individual decision-making process conducted by an interdisciplinary team in consent with the patient's preferences and living conditions.

## Highlights

• Unique case: Unexpected excellent recovery after severe spinal cord injury and traumatic amputation of the upper extremity.
• Both upper limb amputation and spinal cord injury require timely intervention.
• The decision-making process regarding the potential for replantation must consider the overall context of the patient.
• A neurological transition from ASIA A to ASIA D following a Type C fracture is extremely rare.

## 1 Introduction

Advances in microsurgical technique, biomaterials and prosthetics as well as constant refinement of treatment algorithms for polytrauma patients have led to a myriad of new treatment options as well as intricate decisions for present day trauma care units [[1], [2], [3]]. Aside from the intrinsic difficulties of treating a patient with multiple severe injuries there is still considerable debate on the topic of major limb replantation as the functional outcome, especially for the upper extremity, remains challenging to predict [[4], [5], [6]].Therefore, the constant reevaluation of outcomes, especially in the long term is an important aspect in improving the overall treatment of these complex patients and balancing new insights with established concepts.

In our case report we present a 20-year-old patient with polytrauma injuries including severe spinal cord injury as part of an open lumbar spine translational fracture at the level of L1/L2 as well as amputation of the dominant right upper limb at the level of the elbow.

Confronted with the prospect of a young patient with a likely complete paraplegia of the lower extremities and only one functional upper limb, we had to apply an individual decision-making process beyond the established ”life before limb” dogma. It was the challenging task of the trauma care team for this young patient to address the demands of treating his life-threatening injuries while also considering, to the best of our ability, his quality of life in the long term.

## 2 Methods

The case report has been reported in line with the SCARE criteria [30].

Artificial Intelligence has not been used for preparing this manuscript.

## 3 Case report

Under the influence of alcohol, a 20-year-old male patient suffered polytrauma injuries as he fell from a platform of a railway and was hit by a moving train leaving the station.

Paramedics were the first responders at the scene and provided initial treatment with fluid resuscitation, tourniquet and analgesia. In the manner of a “load and go” concept the patient was quickly taken to the ER of our level one trauma center where he presented in stable cardio-pulmonary condition with a GCS of 8 and was rapidly intubated.

**Fig. 1:**
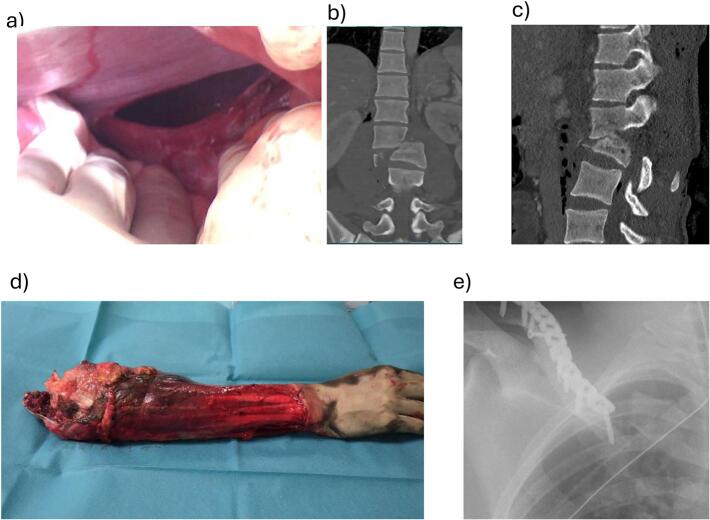
Trauma sequela: a) Diaphragmatic rupture visible in laparotomy, b–c) CT-scan of spinal fracture, d) amputate at occurrence in trauma bay, e) X-ray of peri-implant clavicle fracture.

According to our established polytrauma protocol, a full-body spiral CT scan followed, which revealed bilateral pneumothoraces, lung contusions, a diaphragmatic rupture, an open translocation spinal injury type C at the level of L1/L2, a medial clavicle fracture as well as amputation of the right forearm (Fig. 1 a-d). Fortunately, the cerebral CT imaging was unremarkable, and no intracerebral hemorrhage or skull fracture was detected.

The trauma care team at this point consisted of trauma surgeons, plastic-reconstructive surgeons, vascular surgeons as well as general surgeons and anesthesiologists. The patient continued to show a stable cardio-pulmonary condition und moderate pharmacological support, which allowed for an extensive surgical treatment plan to be formulated while adhering to ATLS™ principles, which was executed as follows:

### 3.1 Pulmonary contusions, pneumothoraces and diaphragmatic rupture

As the most acute injury, initially the chest trauma was addressed. Bilateral chest drains were inserted in typical fashion and a median laparotomy was performed to address the diaphragmatic rupture. Intraoperatively a left diaphragmatic rupture of 8 cm was observed and treated by direct suturing with Ethibond® (J&J Medical, Norderstedt, Germany). No additional intra-abdominal injuries were detected. Rupture or lacerations of the liver or spleen were specifically ruled out.

### 3.2 Replantation of the forearm

Weighing all factors a consensus was reached to attempt replantation of the forearm. This was motivated specifically by a straight separation at the level of the distal humerus with preserved muscles of the forearm and overall moderate contusion and contamination of the soft tissue. In addition, the young patient age and moderately stable condition was taken into consideration and the potential dependency on a wheelchair in the future. Given the level of the injury and the overall status of the patient an arthrodesis at the elbow joint was planned in 30° flexion to allow for bed transfers and wheelchair mobility.

The procedure started approximately 4 h after the injury had taken place and in close coordination with the general and trauma surgeons to minimize surgical delays. Before, the amputated arm was prepared with debridement of bone and clear identification of the median, ulnar and radial nerve as well as the brachial artery, cephalic and basilic veins.

All essential structures showed a sufficient stump length to be considered for reconstruction, so that the replantation was attempted. After identifying the brachial artery and corresponding veins at the level of the upper arm there was no outflow from the artery due to traumatic injury of the vessel intima. In cooperation with vascular surgery a shunt was placed and reperfusion of the forearm was promptly achieved. However, due to the instability of the construct a parallel arthrodesis of the elbow was not possible and after 75 min of reperfusion the shunt had to be removed again with a second ischemia interval.

**Fig. 2:**
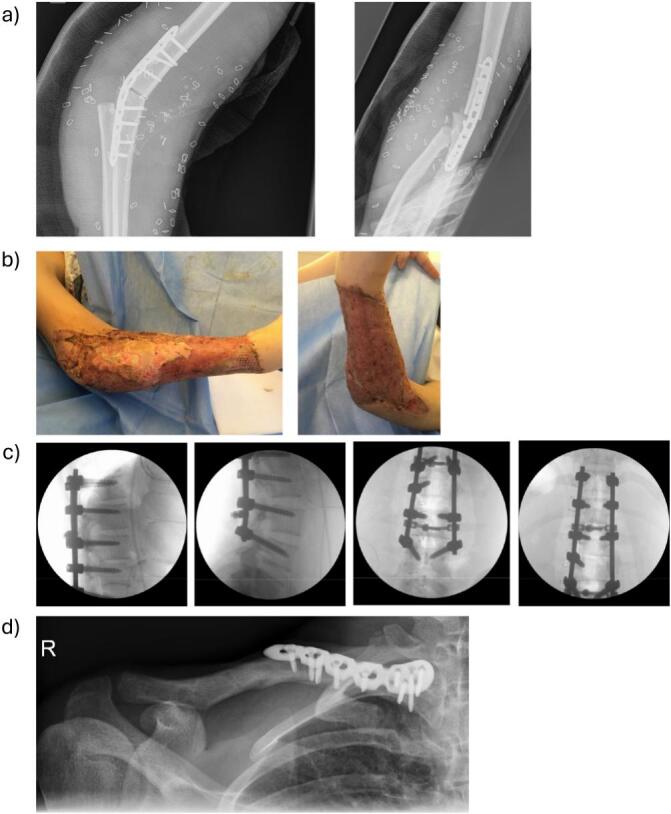
Operative treatment: a) X-ray of elbow-arthrodesis, b) clinical photography after replantation and mesh-grafting, c) fluoroscopy of dorsal instrumentation, d) X-ray of plate osteosynthesis of peri-implant clavicle fracture.

Arthrodesis was then performed between the forearm and upper arm, after bony resection, using a 3.5 mm 10-hole LCP plate fixation (Fig. 2a) at an angle of approx. 30°. Consecutively, anastomosis of the brachial artery was performed under the surgical microscope with 8.0 monofilament suture and four coupler-anastomoses (2 × 3,5 mm and 2 × 2,5 mm) between two brachial veins and two branches of the cephalic/basilic veins were carried out. Lastly the median nerve, ulnar nerve and radial nerve were coapted with epineural 8.0 monofilament sutures and additionally braced by fibrin glue, after shortening the end on the amputated parts close to the muscle to minimize length of reinnervation.

To address the anticipated ischemia reperfusion injury of the right forearm, an extended carpal tunnel release and compartment release of the ventral forearm for both, the superficial and deep flexor interval, was performed. The joint origin of superficial and deep flexor muscles was reinserted at the level of the distal humerus with sutures to the periosteum. The biceps muscle and brachialis muscle were also sutured to the periosteum of the radius and the triceps muscle was sutured to the arthrodesis plate posteriorly. Finally, the defect was covered with a full-thickness skin graft of the right forearm decollement and temporary coverage with EpiGARD® (Biovision, Ilmenau, Germany).

Specific postoperative medication included Liquemin 10,000 IE / 24 h i.v. starting 6 h after the surgery, oral medication using Aspirin 1 × 100 mg, and i.v. antibiotic treatment using combination of amoxicillin and clavulanic acid 3 × 2.2 g/ per day.

Due to partial necrosis of the full-thickness skin graft a revision surgery had to be undertaken on postoperative day 8. The skin graft was partially debrided including superficial parts of necrotic muscle and the defect was covered with a random pattern lateral arm flap. The donor site was covered with split thickness skin grafting from the upper thigh. A final split-thickness skin graft was required on postoperative day 21 due to insufficient healing result at the flap donor site (Fig. 2b).

### 3.3 Treatment of the L1/ L2 spinal fracture

Throughout the surgery the patient continued to destabilize with hypotonic blood pressure, loss of blood volume as well as worsening coagulopathy. The decision was therefore made to stop surgical treatment after replantation of the arm to stabilize the patient. Following the extended procedure the patient remained intubated and ventilated and was taken to the ICU. One day after trauma we saw a sufficient recovery to then treat the spinal fractures.

Initially, dorsal spondylodesis of T10 to L3 and laminectomy of L1-L2 was performed (Fig. 2c). One month following dorsal instrumentation ventral spondylodesis and fusion of the vertebral bodies L1 and L2 using the Medtronic legacy anterior 6.5x45mm system (Medtronic, Dublin, Ireland) was carried out.

### 3.4 Surgical treatment of the clavicle fracture

The peri-implant clavicle fracture on the right side was treated 2.5 weeks after the initial trauma. The clavicle plate, which had been inserted for treatment of a right clavicle fracture one year prior to the current polytrauma and amputation injury, was removed and osteosynthesis was performed using an anatomic 4-hole superior-anterior 2.7/3.5 mm LCP (Fig. 2d).

### 3.5 Follow-up treatment and further course

After completion of all surgical treatment, the patient was finally discharged to a rehabilitation facility 6 weeks after trauma. On discharge, the neurological status of the lower extremities demonstrated an ASIA A score. The initial post-traumatic and preoperative status could not be determined, as the patient was intubated immediately after arrival in the ER and no active movements of patient extremities was observed. While it was therefore assumed that complete paraplegia was already existent posttraumatic, this cannot be guaranteed with absolute certainty.

Surprisingly, the patient's neurological status improved under intensive physiotherapeutic and rehabilitative care. Just 9 months post injury, the ASIA score improved to C, with active movements in the hip flexors and knee extensors against gravity (M2/5). This correlates with level L2-L3. Two and a half years postoperatively, the value ameliorated to ASIA D, and muscle strength demonstrated values ranging from M3–5/5 for specific muscles of the L2, L3 and S1 segments. The radiological course of the spinal fractures was also positive, with good consolidation of the spinal fractures without secondary fracture collapse at the 10 month and 5-year posttraumatic radiologic follow up.

**Fig. 3:**
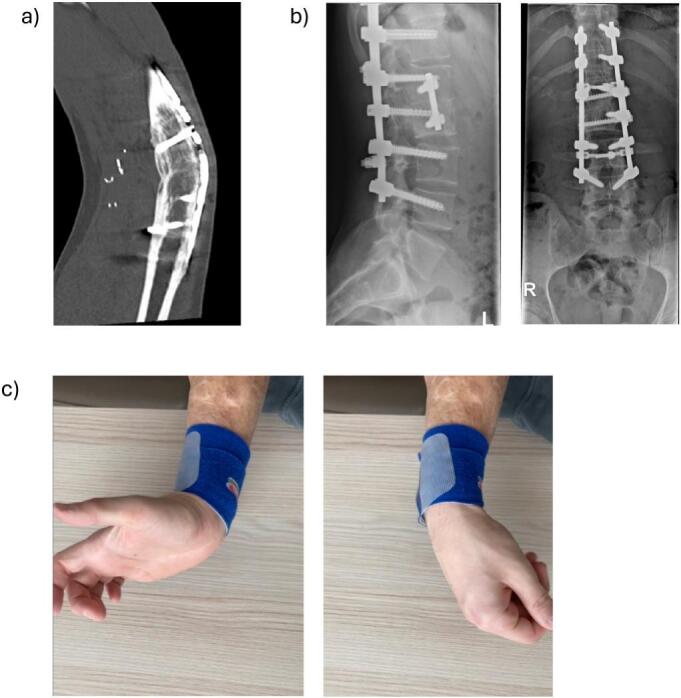
Outcome after 5 years: a) CT-scan of healed elbow-arthrodesis, b) X-ray of healed spinal fracture, c) clinical photography of dorsal and plantarflexion in right wrist.

In the area of the elbow arthrodesis, the soft tissue healing progressed without complication during the rehabilitation process. However, bony consolidation was delayed, with the patient experiencing pain up to two and a half years postoperatively and still incomplete osseous consolidation at that time. In our opinion a surgical revision was not recommended given the risks for sever complications. Finally, at the 5-year follow up, complete osseous consolidation was observed (Fig. 3a-b) and symptoms of pain and discomfort had subsided.

Lastly the function of the right arm had progressed far beyond our expectation and the patient demonstrated full closure of the fist and full finger extension with a strength of M5/5 as well as flexion and extension in the wrist with M5/5 (Fig. 3c, Video 1). Grip strength on the right showed 5 kg versus 23 kg on the left.

The sensory recovery showed a two-point-discrimination of 8 mm from thumb to ring finger and 15 mm for the little finger. Due to the elbow arthrodesis, function of pronation and supination was expectedly significantly impaired. However, this could be compensated by shoulder and upper body movements to an adjusted 70° pronation/supination.

In summery 5 years after the operation, the patient had resumed many activities in daily life and was coping well with some challenging aspects of living at home. Neurological improvements continued and the patient was able to walk independently despite persistent foot drop paresis, which was treated using a Heidelberg splint. Intermittently, the patient utilized a wheelchair for longer walking distances. He also mastered successful reintegration into his previous profession. The patient is satisfied with the course of his recovery and describes good integration in his social life, even though minor disabilities exist, for which he developed several coping strategies.

## 4 Discussion

Polytrauma is a challenging situation for the healthcare team and requires an interdisciplinary treatment strategy. According to the ATLS™ guideline, acute life-threatening injuries must be addressed before peripheral injury patterns are treated. In this case, thoracic trauma was one of the acute life-threatening injuries. After insertion of bilateral chest tubes to ensure stable airway, breathing and cardiac conditions, explorative laparotomy and suturing of the diaphragmatic rupture was performed. Subsequently, therapeutic strategy focused on treatment of the amputation of the upper limb prior to spinal instrumentation, for which we had to consider a multitude of deciding factors.

In a polytrauma case with life threatening injuries and an unstable patient condition, traumatic amputation of a major extremity can be accepted and according to damage control guidelines acute soft tissue management and stump formation may preferentially be performed.

In addition, prognosis for successful macro-replantation with a good functional outcome is difficult and on average three follow-up operations are required [6]. Also an extended time of healing and recovery of 5.4 years on average has been reported until satisfactory results are achieved [5]. The risk of daunting results with chronic pain and overall unacceptable long-term functional outcome with patients requesting a secondary resection of replanted extremity are not to be taken lightly in the face of ambitious surgical goals.

Thus, it is always important to carefully evaluate each case individually and discuss, if possible, with the patient the prognosis and expectations prior to replantation so that he or she may engage in the decision – making process [7]. A comprehensive assessment should reflect the overall prognosis, including potential complications and long-term outcomes, which is essential in the decision-making process.

The prognosis for replantation of a major extremity amputation depends on several factors including the level of amputation, the condition of the amputated limb, the skill of the surgical team, and the overall health of the patient [8].

To date there is unfortunately still no scoring system established with an acceptable high predictive value in patient functional outcome [8]. Many systems like the Mangled Extremity Severity Score (MESS) lack a sufficient predictive value in this regard and a team approach with experienced surgeons is often recommended [9].

In our experience congruent with the current literature a few major factors are worthwhile considering in such difficult situations.

First and foremost a key for successful macro replantation is rapid surgical intervention [10]. The longer the period between injury and replantation, the higher the risk of tissue necrosis and failure. Ideally reperfusion can be achieved within 6 h of the traumatic amputation [11]. In the context of blood supply the status of blood vessels and the soft tissue cannot be neglected. Crush injuries or sever contusion of soft tissue and blood vessels may severely compromise survival of the limb despite flawless anastomosis.

While a prompt primary vessel repair is the goal to strive for, it usually requires a lengthy preparation of vessels and sometimes additional reconstructive techniques like autografts or allografts are needed.

A valuable resource worth considering in such situations is temporary intravascular shunting (TIVS), as used in this patient. The concept of placing bridging tubes to minimize ischemia time was described as early as 1915 and has repeated mentioning in the literature thereafter [12]. However, this is often regarding combat injuries suffered in the military, while its use in the civilian sector is less frequently described [13]. Current data show a decreased risk of compartment syndrome with the placement of TIVS [14], but interestingly there is little data to compare limb salvage success and shunting, especially regarding the upper extremity. We believe that in our case it was nonetheless a key maneuver to prevent further ischemic damage and aided in the positive outcome.

Beyond a viable tissue recovery, we aimed for a functional recovery in upper limb replantation, which surely poses one of the greatest challenges in any such reconstructive endeavor.

While nowadays the repertoire of reconstructive surgery is broad regarding muscle transplants and tendon transfers, the reconstruction and regeneration of peripheral nerves is a topic of intense research and ongoing changes in clinical practice.

In the 1980s functional recovery after an amputation at the level of the upper arm were often limited to some elbow motor function as well as protective sensitivity at the level of the hand. Children and adolescents showed slightly more promising results, however, still with severe functional deficits [[15], [16], [17]]. Looking at the literature today we dare to be slightly more optimistic. In a recent study undertaking a systematic review looking at 301 patients with replanted arms, satisfactory outcome could be achieved for about 40 % above the elbow, 55 % through the elbow and 50 % below the elbow [18]. A review from 2020 reported 50 % of cases with a Chen Score of I or II in an analysis of 79 replanted Arms [19].

It is established that the muscles supplied by the radialis nerve show the least potential for recovery where the finger flexors supplied by the median nerve show the best outcomes. Despite improvements in nerve grafting and repair a few limiting factors remain to date. The growth rate of human peripheral nerves of about 1 mm/day must be considered as well as the degeneration of muscle over time and replacement with fat tissue. In the here reported case the patient had the benefit of relatively long proximal nerve stumps, which had to bridge a smaller distance to the target muscle as would have been expected given the level of the amputation. None the less the recovery observed here can be seen as exceptional.

A second factor that should be considered with special regard to nerve recovery is age as it affects neuroplasticity. Aside from the reestablishment of the peripheral circuitry there are also many changes at the level of the central nervous system that impact the functional end result [20]. Therefore, it is one of the many reasons a young patient should receive special attention in these circumstances.

Furthermore, the condition of the amputated part, including tissue viability and extent of injury, determines the feasibility of replantation. If the severed part is grossly contaminated, severely damaged or mangled replantation may not be possible or advisable [21]. In the same context soft tissue coverage is necessary to support the replanted part and prevent infection but thankfully nowadays multiple options of temporary wound coverage exist, with resulting good feasibility of potential multistage soft tissue reconstruction if necessary.

Not to be overlooked is the overall health and medical history of the patient regarding feasibility of the surgery in the first place and with respect to the success of replantation. Factors such as age, smoking status, presence of chronic diseases (e.g., diabetes), and nutritional status can all impact wound healing and recovery [21]. Furthermore, replantation should be considered in the context of the patient's functional goals and expectations post-surgery. This includes the patient's occupation, lifestyle, and desired level of function. For example, replantation may be indicated for amputations involving the hand, fingers, thumb, foot, or toes, where preserving motor function and sensation is critical for daily activities and quality of life. Linked to the functional goals, patient's age plays a major role as well. Studies showed that functional results in patients aged over 50 years are often insufficient [8,22] Aftercare and Rehabilitation also play a crucial role in the final functional outcome. Patients require intensive physical therapy and occupational therapy to regain function and adapt to any functional limitations resulting from the injury [17]. Individual outcomes may vary, and it is essential for patients to have realistic expectations and actively participate in their recovery process [23].

In summary, positive predictors for possible good outcome of macro- replantation included patient age, good overall health conditions, as well as a short ischemia time and early replantation in a level one trauma center with trauma-, vascular- and plastic surgery expertise. Furthermore, status of soft tissues and vascular status must be acceptable.

In addition to these parameters, decision to proceed with macro-replantation in our case was strongly influenced by the fracture morphology of the spinal trauma and anticipated paraplegia due to bony destruction and dislocation of the spinal canal at the level of L1/L2 although no precise neurological evaluation or ASIA score was obtained preoperatively due to primary sedation and consecutive early intubation of the patient. From the radiological assessment we concluded that paraplegia would result in dependency on a wheelchair in the long term.

Thus, a second upper limb was obviously of paramount importance for the patient to be able to push a wheelchair independently and effectively. An arthrodesis angle of 30° was chosen, to ensure ideal use of the wheelchair, contrary to 45–110° commonly suggest in literature as fusion angle [24].

A critical but necessary delay had to be placed on the treatment of the above-mentioned spinal fracture. While this injury was also severe it was placed below the arm replantation in the surgical hierarchy and in the end had to be delayed by one day due to unstable condition of the patient. The general recommendation in spinal injuries with neurological deficits is treatment within the first 24 h [25,26], which was still achieved by a small margin and completed in the preferred manner with extended dorsal instrumentation followed by additional and sequential ventral stabilization and spondylodesis.

Even with meticulously adherence to these guidelines, the neurological progression in our patient exceeded expectations for both the spinal injury as well as injury of the upper limb. The frequent long-term sequelae of paraplegia included urological problems such as bladder emptying disorders, urinary retention, and urinary tract infections. Orthopedic-static problems such as progressive spinal misalignment, contractures and spontaneous fractures in osteoporosis were also reported. The transition from postoperative ASIA A to ASIA D is described in the literature as extremely rare. Even the progression from ASIA A to ASIA B occurs in less than 20 % of cases [27]. A functionally significant recovery has only been reported in singular cases [28]. However, a limitation of this case report is, that the ASIA score was not obtained directly after trauma and posttraumatic sensomotoric status can therefore only be assumed in the clinical context.

Also as mentioned earlier the chances of a satisfactory result of arm replantation at the elbow-level, as reported by patient questionnaires, is only up to 55 % in a mixed cohort [18]. Similarly using Chen's criteria for evaluation of a functional result we see a large spread in reports from 30 % to 60 % with a score of 1 or 2 [19].

In addition, one must consider the many advances, which have been made in prosthetics with myoelectric prosthesis at the forefront of good functional results [18]. Nonetheless the challenges of careful patient selection, availability, financial investment and long training intervals should not be neglected. Currently we see similar satisfaction with such devices when the injury was suffered at the level of the upper arm or elbow [8].

Aside from precise surgical technique by experienced surgeons in a multidisciplinary team we think this case shines new light on the debate to salvage limbs in young patients. The potential for superior healing potential in these cases regarding peripheral nerve recovery has been documented before [29] and we believe that a mixture of local healing processes as well as central neuroplasticity lie the foundation for extraordinary results as observed in this case.

## 5 Conclusion

Here we report a rare case of an excellent recovery after spinal cord trauma and macro-replantation of traumatic amputation of the upper limb at the level of the elbow. The decision to replant the limb was made in context of the young patient age and to enable the patient to reintegrate into life with independent wheelchair mobility due to suspected traumatic paraplegia as well as considering numerous predictors by an experienced surgical team.

Remarkably the neurological recovery has exceeded far beyond our expectations and surpassed many results reported in the current literature to a functional level of mobility independent of a wheelchair and active wrist as well as finger movements with strength M5/5.

We hope to further aid in the refinement of complex treatment plans and decision-making processes to generate best possible outcomes for critically injured patients.

The following is the supplementary data related to this article.

Video 1 Wrist movement 5 years postoperative. Video 1

## Informed consent

Written informed consent was obtained from the patient for publication of this case report and accompanying images. A copy of the written consent is available for review by the Editor-in-Chief of this journal on request.

## Ethical approval

Informed consent was obtained. Further ethical approval was not necessary for the case report. The case report was conducted at University Hospital Zurich.

Written informed consent was obtained from the patient. No further ethical approval was necessary.

## Guarantor

Christian Hierholzer.

## Research registration number

No clinical study was performed.

## Funding

No funding was received for this case report.

## Author contribution

CH, CTH, HCP, TM and NL did clinical investigation and surgery on the patient. CH and NL supervised the study. CTH, HCP, VS and NL did literature research. CTH, VS and CH wrote the manuscript. All authors provided critical feedback and proof-reading of the manuscript.

## Declaration of competing interest

There are no conflicts of interest to be reported by any of the authors.
